# Treatment of Phthisis Pulmonalis

**Published:** 1857-12

**Authors:** 


### TREATMENT OF PHTHISIS PULMONALIS.

Mr. Churchill presented to the Acadamie de Medicine of Paris the following *resume* of a memoir on the cause and treatment of consumption: "The total number of cases treated by me were thirty-five, all of which had advanced to the second or third stage, that is to say, the tubercles were either undergoing softening, or had been more or less evacuated, leaving cavities behind. Of this number, nine were completely cured, and in eight of the nine the physical signs have entirely disappeared; eleven more cases were very much improved, fourteen died, and one is under treatment.

"The immediate cause, or at least an essential condition of the tuberculous diathesis, is the diminution in the economy of oxygenizable phosphorus. The specific remedy of this disease consists in a preparation that has the double property of easy assimilation and oxydation. The hypophosphites of soda and lime are the preparations which appear thus far to unite the two conditions. Administered in doses varying from ten to sixty grains a day, they may be employed indifferently or alternately in the treatment of phthisis. The maximum dose which I have administered to the adult is twenty grains. They produce an immediate effect upon this diathesis, and cause to disappear with marvelous rapidity all the symptoms which express the phthisical condition.

"When the deposit which results from this diathesis is recent, when the softening is but just commenced, when these processes do not advance too rapidly, the tubercles are re-absorbed, and disappear without leaving a trace behind. When the deposit is older, when the softening has attained to a certain degree, it continues sometimes in spite of treatment. The issue of the disease depends upon the anatomical lesion, its extent, and above all, the presence or absence of complications.

"Of the numerous trials I have made with various substances used by inhalation, I have seen no good results from them in modifying salutarily the local lesions.

"The hypophosphites of soda and lime are *certain* prophylactics against tubercular diseases. The physiological phenomena I have observed during the administration of the hypophosphites of soda, lime, potassa and ammonia, show that these preparations have a double action. On the one hand, they augment the principle that generates nerve force; on the other, they are *par excellence* superior to every other known hematogene. They possess in the highest degree the therapeutic properties attributed by the ancients to phosphorus, without one of the dangers which caused this latter substance to fall into disrepute. There is no doubt but that the hypophosphites will occupy at no distant day the front rank among therapeutic agents."—*Archives Generale de Medicine.*

